# A case report and literature review: previously excluded tuberculosis masked by amiodarone induced lung injury

**DOI:** 10.1186/s40360-018-0279-1

**Published:** 2018-12-29

**Authors:** Egle Karinauske, Silvijus Abramavicius, Greta Musteikiene, Edgaras Stankevicius, Jurgita Zaveckiene, Vidas Pilvinis, Edmundas Kadusevicius

**Affiliations:** 1 Institute of Physiology and Pharmacology, Lithuanian University of Health Sciences, A. Mickeviciaus str. 9, 44307 Kaunas, LT Lithuania; 2 Department of Pulmonology, Medical Academy, Lithuanian University of Health Sciences Kaunas Clinics, Kaunas, Lithuania; 3 Department of Radiology, Medical Academy, Lithuanian University of Health Sciences Kaunas Clinics, Kaunas, Lithuania; 4 Department of Intensive Care, Medical Academy, Lithuanian University of Health Sciences Kaunas Clinics, Kaunas, Lithuania; 5 Intensive care unit, Republican Vilnius University Hospital, Vilnius, Lithuania

**Keywords:** Clinical pharmacology, Amiodarone induced pulmonary toxicity, Atrial fibrillation, Adverse drug reaction, Polypharmacy

## Abstract

**Background:**

Amiodarone is an antiarrhythmic drug which is used to treat and prevent several dysrhythmias. This includes ventricular tachycardia and fibrillation, wide complex tachycardia, as well as atrial fibrillation (AF) and paroxysmal supraventricular tachycardia. Amiodarone may prove to be the agent of choice where the patient is hemodynamically unstable and unsuitable for direct current (DC) cardioversion. Although, it is not recommended for long-term use. The physician might encounter issues when differentiating amiodarone-induced lung toxicity with suspicion of interstitial lung disease, cancer or vasculitis. Adverse drug reactions are difficult to confirm and it leads to serious problems of pharmacotherapy.

**Case presentation:**

A 78-year-old Caucasian male pensioner complaining of fever, dyspnea, malaise, non-productive cough, fatigue, weight loss, diagnosed with acute respiratory failure with a 16-year long history of amiodarone use and histologically confirmed temporal arteritis with long-term glucocorticosteroid (GCC) therapy.

Patient was treated for temporal arteritis with GCC for ~ 1 year, then fever and dyspnea occurred, and the patient was hospitalized for treatment of bilateral pneumonia. Chest X-ray and chest high resolution computed tomography (HRCT) indicated several possible diagnoses: drug-induced interstitial lung disease, autoimmune interstitial lung disease, previously excluded pulmonary TB. Amiodarone was discontinued. Antibiotic therapy for bilateral pneumonia was started. Fiberoptic bronchoscopy with bronchial washings and brushings was performed. Acid fast bacilli (AFB) were found on Ziehl-Nielsen microscopy and tuberculosis (TB) was confirmed (later confirmed to be *Mycobacterium tuberculosis* in culture), initial treatment for TB was started. After a few months of treating for TB, patient was diagnosed with pneumonia and sepsis, empiric antibiotic therapy was prescribed.

After reevaluation and M. Tuberculosis identification, the patient was referred to the Tuberculosis hospital for further treatment. After 6 months of TB treatment, pneumonia occurred which was complicated by sepsis. Despite the treatment, multiple organ dysfunction syndrome evolved and patient died. Probable cause of death: pneumonia and sepsis.

**Conclusions:**

The current clinical case emphasizes issues that a physician may encounter in the differential diagnostics of amiodarone-induced lung toxicity with other lung diseases.

## Background

Amiodarone is an antiarrhythmic drug which is used to treat and prevent several dysrhythmias. This includes ventricular tachycardia and fibrillation, wide complex tachycardia, as well as atrial fibrillation (AF) and paroxysmal supraventricular tachycardia. Amiodarone may prove be to the agent of choice where the patient is hemodynamically unstable and unsuitable for direct current (DC) cardioversion. It is recommended in such a role by the UK government’s National Institute for Health and Clinical Excellence (NICE) [1] and American College of Cardiology, American Heart Association and Heart Rhythm Society [2]. Although, it is not recommended for long-term use. One of amiodarone adverse events is drug-induced respiratory disease (DIRD) which is rare and can be subdivided into drug induced pulmonary parenchymal disease [3] and drug-induced interstitial lung disease (DILD) [4]. DILD may present when using amiodarone and other medicines, such as angiotensin converting enzyme inhibitors (ACEi), angiotensin receptor blockers (ARB), anticoagulants, aspirin, beta blockers (BB) etc. [5]. Patients with DILD can present with a number of symptoms, such as progressive shortness of breath (dyspnea), non-productive cough, malaise, fever, pleuritic chest pain (Table 1), and it is important to ascertain the duration, severity, and progression of symptoms. Chest X-ray may show radiological abnormalities, such as reticular, nodular or mixed patterns [6]. The images of the chest high-resolution computed tomography (HRCT) usually contain nonspecific findings including patchy, scattered or diffuse ground-glass opacities, consolidation, irregular reticular opacities and fibrosis (traction bronchiectasis, honeycombing) [7].

**Table 1:** Possible differential diagnosis of lung diseases in the current case history

Possible diagnosis	Clinical symptoms	Radiological findings
Interstitial lung disease	Dyspnea, non-productive cough, malaise, fatigue, weight loss	X-ray – consolidation, fibrosis; HRCT – consolidation, fibrosis, ground glass partial alveolar filing, reticulonodular pattern.
Tuberculosis	Productive cough, malaise, fatigue, weight loss, night sweats, hemoptysis	X-ray – infiltration, cavitation, nodularity, hilar/paratracheal lymphadenopathy, pleural effusion, atelectasis; HRCT – infiltration, granulomas and tree-in-bud appearance
Amiodarone-induced interstitial lung disease	Progressive shortness of breath (dyspnea), non-productive cough, malaise, fever, pleuritic chest pain	X-ray – consolidation, fibrosis; HRCT – diffuse interstitial pneumonitis with fibrosis and ‘ground-glass’ opacities, consolidation
Vasculitis	Fever, weight-loss, fatigue, evidence of multisystem involvement, rashes	X-ray – pneumonia-like x-ray picture; HRCT – bilateral perihilar or peripheral ground-glass opacities, pulmonary haemorrhage
Wegener’s granulomatosis	Necrotizing granulomatous lesions of respiratory tract, ulcers, malaise, fatigue, weight loss	HRCT – perihilar or peripherical ground-glass opacities, pulmonary haemorrhage, necrotizing granulomas
Lung tumor	Dyspnea, non-productive cough, malaise, fatigue, weight loss, hemoptysis	X-ray – nodule or mass with hilar enlargement, lobulated hilar mass, atelectasis; HRCT – solid or mixed pulmonary nodules or mass, atelectasis, lymphadenopathy
Bacterial lung infections	Fever, chills, productive cough, dyspnea, pleuritic chest pain, fatigue	X-ray – consolidation of the lobe, dense opacities, pneumothorax, hydrothorax; HRCT – centrilobular nodules, tree-in-bud pattern, pleural-based consolidation

Underlined symptoms are suitable to the patient

The chest HRCT provides greater diagnostic accuracy than the plain chest radiograph and helps to narrow the differential diagnosis of interstitial lung disease (ILD). For example, bilateral symmetric hilar adenopathy and upper lung zone reticular opacities suggest sarcoidosis or another granulomatous disease; or in an asymptomatic patient, diffuse, calcified, nodular, interstitial opacities may reflect healed varicella-zoster pneumonia [8].

Histologically DILD may be identified as interstitial pneumonia (an inflammation of the lung interstitium, such as alveolar septa), hypersensitivity pneumonitis (the interstitial space is infiltrated by lymphocytes and plasma cells), bronchiolitis obliterans, organizing pneumonia and other [9]. Amiodarone acts via cytotoxic T cells and immunological reaction resulting in a diffuse interstitial pneumonitis with fibrosis and ‘ground-glass’ opacities in chest HRCT [10].

It is important to keep in mind the other possible pulmonary diseases to differentiate properly. One of them is tuberculosis (TB) which remains an important health problem in Lithuania with ~ 41.89 new TB cases per 100,000 population (2015) [11]. Thus, the specificity of BACTEC is ~ 99% [12], there are cases when TB is not diagnosed because of low concentration of bacteria in the sputum or bronchial washings. The other important differential diagnosis are ILD which might look very similar to DILD on HRCT scans, vasculitis, Wegener’s granulomatosis, lung cancer and other bacterial infections.

We present a case of acute respiratory failure, with previously excluded TB (based on negative bacteriological testing for TB), amiodarone use (16 years 400 mg/daily) and histologically confirmed temporal vasculitis with suspected exacerbation. In accordance with patient history and the radiological findings the differential diagnosis in this case included the drug-induced pulmonary toxicity, autoimmune lung injury, TB and other lung diseases suitable to this case.

## Case presentation

A 78-year-old Caucasian male was diagnosed with temporal arteritis based on clinical presentation and biopsy of temporal artery showing granulomatous inflammation. The chest HRCT revealed pulmonary changes similar to autoimmune ILD. Based on radiological findings, the diagnosis of pulmonary TB was considered but sputum smears and microscopy of bronchial washings were negative and this diagnosis was rejected. Thus, the treatment with methylprednisolone (8 mg/daily) for temporal arteritis was initiated more than a half-a-year ago. At the time, patient was using amiodarone tablets 400 mg/daily for recurrent atrial fibrillation (AF) in 2002.

Patient arrived at the rheumatology outpatient department, complaining of fever and dyspnea, his parameters of central hemodynamics were normal, ECG showed sinus rhythm, C-reactive protein (CRP) was 118 mg/l. The diagnosis of bilateral pneumonia, consistent with pulmonary vasculitis, was established with chest X-ray, and patient was admitted to the rheumatology department. Empiric antibiotic therapy with cefuroxime (4.5 g/daily) and methylprednisolone (32 mg/daily) for suspected exacerbation of vasculitis were prescribed.

**Fig. 1:**
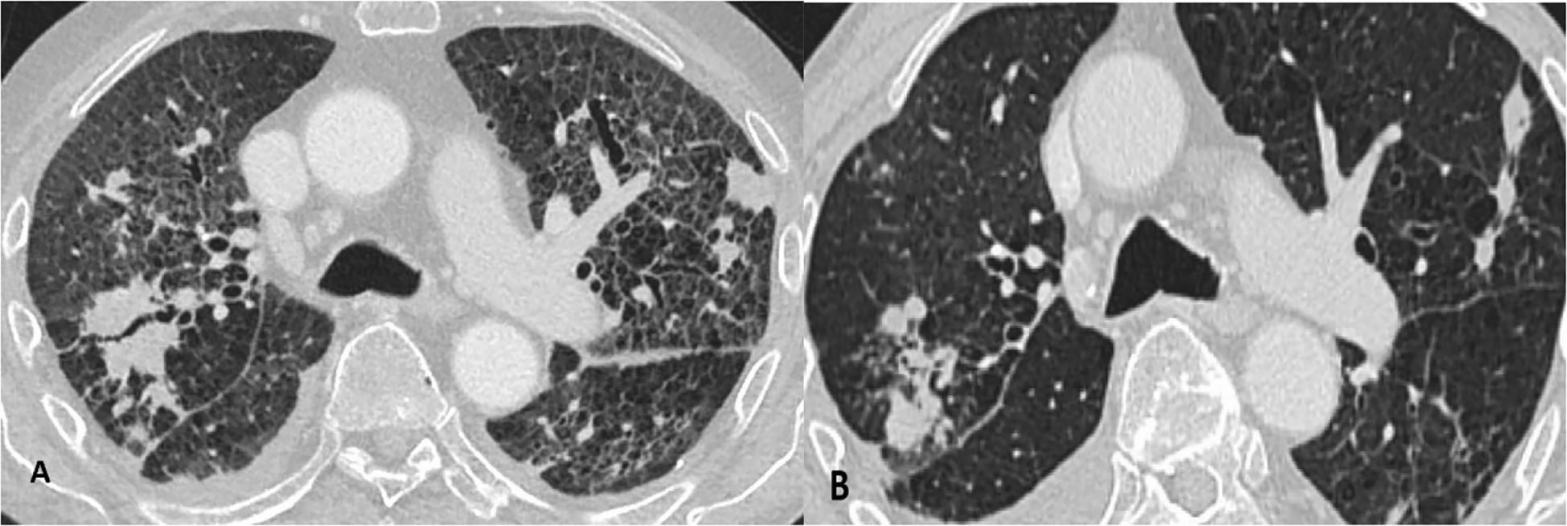
Axial reconstruction of chest HRCT before (**a**) and after (**b**) the treatment

**Fig. 2:**
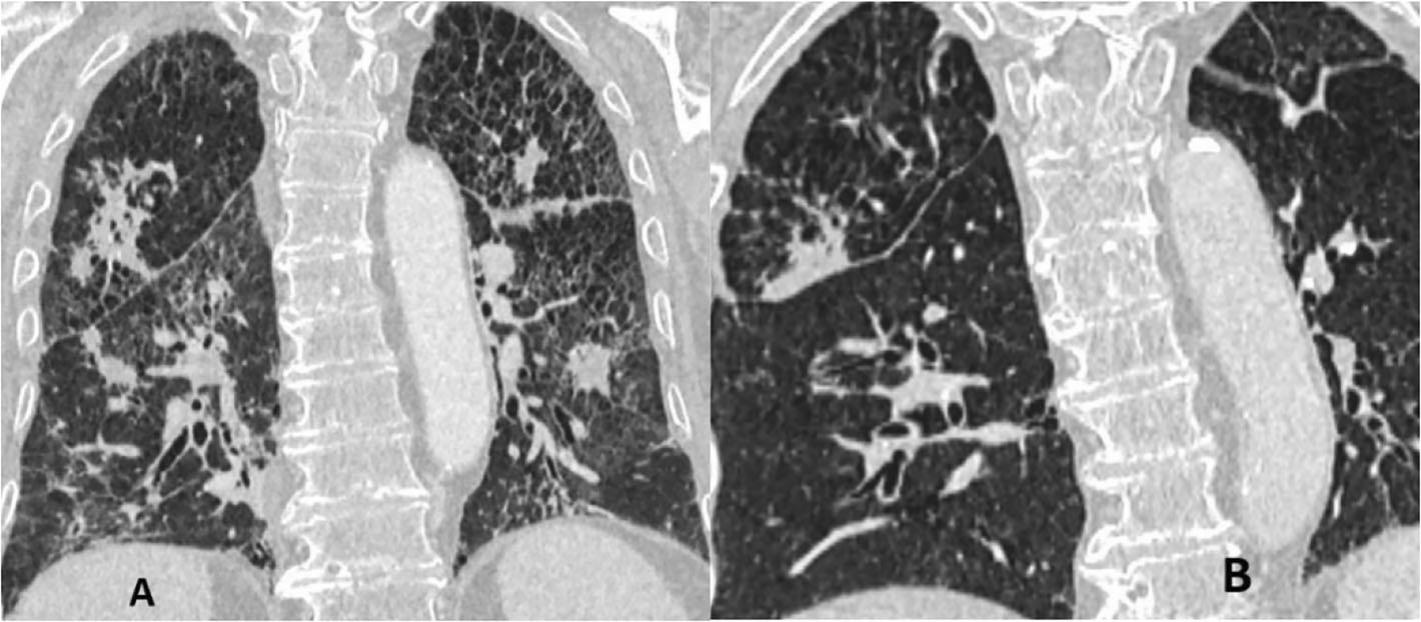
Coronal reconstruction of chest HRCT before (**a**) and after (**b**) the treatment

**Fig. 3:**
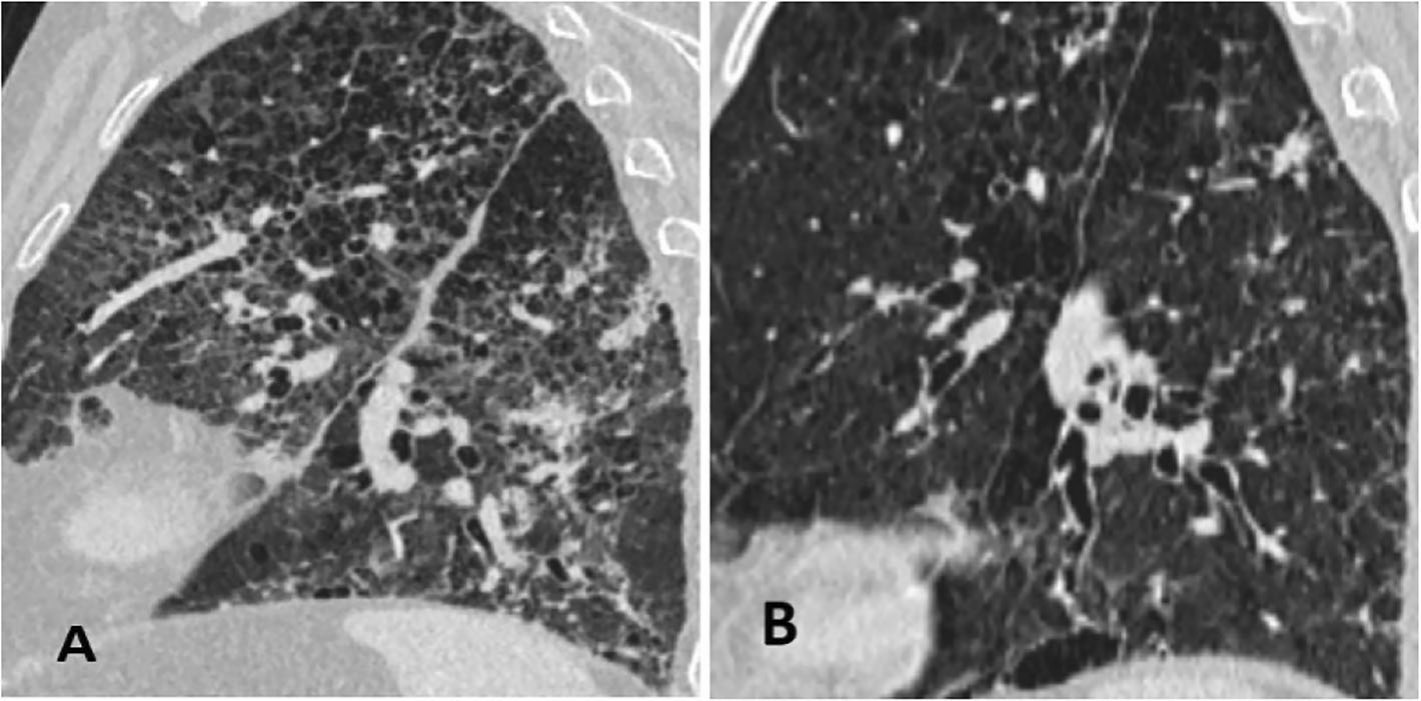
Sagittal reconstruction of chest HRCT before (**a**) and after (**b**) the treatment

On the third day of hospitalization, patient complained of malaise, pressure and tightness in his chest. ECG was performed and the AF was identified. The patient was transferred to the Intensive Care Unit (ICU) for direct current cardioversion into the sinus rhythm. Later the patient was successfully transferred back to the rheumatology department. After 24 h, another episode of AF recurred. Electrolytes were within normal range. Pharmacological conversion was prescribed with 300 mg amiodarone IV solution. Despite the pharmacological management of the AF, patient presented with severe dyspnea, tachypnea with signs respiratory insufficiency. The multidisciplinary consult concluded that the patient suffers either from autoimmune interstitial lung disease or drug induced lung injury. In addition, there was little evidence to exclude bacterial infection. Thus, it was decided to discontinue amiodarone therapy, initiate corticosteroid therapy, and repeat bronchoscopy, take sputum smears and bronchial washings for bacterial diagnostics and chest HRCT. The chest HRCT identified pulmonary emphysema, thickening of intra- and interlobular septa, with random distribution nodules, ground-glass opacities, fibrosis and traction bronchiectasis, progression of mediastinal lymphadenopathy (Figs. 1a, 2a and 3a). Fiberoptic bronchoscopy with bronchial washings and brushings was performed. Acid fast bacilli (AFB) were found on Ziehl-Nielsen microscopy. The diagnosis of TB was confirmed and the anti-TB therapy was initiated with isoniazid (300 mg/day), rifampicine (600 mg/day), ethambutol (1200 mg/day) and pyrazinamide (2000 mg/day). Control chest HRCT was performed after four months of TB treatment. Signs described on earlier chest HRCT regressed – nodules became more restricted with thinning of intra- and interlobular septa shown (Figs. 1b, 2b and 3b). However, the patient developed pneumonia. Blood culture did not confirm bacterial growth, but sepsis was diagnosed clinically. Empiric antibiotic therapy was prescribed. Despite the treatment, multiple organ dysfunction syndrome evolved and patient died. The lung biopsy was not performed thus it is not possible to completely exclude amiodarone induced interstitial lung disease accompanying pulmonary TB.

## Discussion

The current clinical case shows that there are many possible lung diseases that should be taken into consideration when looking for the cause of acute respiratory failure (Table 1). DILD may evolve if untreated as a typically nonnecrotising granulomatous lung disease, hilar or mediastinal lymphadenopathy may be present on chest HRCT [13]. Depending on the immune status of patients, special stains and molecular analyses are necessary to differentiate DILD from mycobacterial or other infections. A lot of drugs (methotrexate, interferon, amiodarone, infliximab, etanercept, leflunomide, mesalamine and sirolimus) can be causative of granulomas in drug-induced respiratory diseases [14]. The current patients’ chest HRCT showed signs suitable to drug-induced ILD (Figs. 1a, 2a and 3a). Amiodarone pulmonary toxicity should be taken into consideration, especially in elderly patients with respiratory symptoms and pulmonary changes, even if only a low dose of amiodarone is administered over a longer time period [15]. The current patient was using amiodarone for AF at least for 16 years 400 mg/daily. It is known that amiodarone tends to accumulate in fatty tissues and its elimination half-life is 25 to 100 days, pulmonary toxicity may initially progress despite drug discontinuation and may recur upon steroid withdrawal [16]. In addition, the diagnosis of TB does not exclude the possibility of accompanying amiodarone toxicity. The correct way to exclude drug-induced ILD is to perform an open lung biopsy (OLB), however, these findings are not always pathognomonic for drug toxicity and correlation with clinical, laboratory, and radiologic data is required. Thus OLB may aid the exclusion of underlying autoimmune disease or infection and document the pattern of lung injury. Another approach is to perform the transbronchial biopsy, but it has more limitations because the lung tissue obtained is very small and may be non-specific [17]. It was found that HRCT performed without intravenous contrast shows increased density in parenchymal organs, such as lungs and liver, when using amiodarone [18]. So the TB and autoimmune ILD were other of many possible explanations based on clinical grounds and patient‘s history. The diagnosis of TB was considered because of the frequency of this illness in Lithuania and findings on chest HRCT. There were only nonspecific clinical signs, such as prolonged cough, lymphadenopathy, fever, night sweats and weight loss, without typical radiological findings for TB like focal infiltration of the upper lobe(s), cavitation, tissue destruction, and fibrosis with traction bronchiectasis, enlargement of hilar/mediastinal lymph nodes, small nodular lesions and pleural effusions [19]. In the current case radiological findings did not show any specific TB changes (Figs. 1a, 2a and 3a). It should be considered that TB may also present with nonnecrotising granulomas on chest HRCT, depending on the immune status of the patient [13]. Immunosuppressive treatment may cause complications, such as bacterial infections. In the current case the patient suffered from pneumonia which was complicated by sepsis with multiple organ dysfunction syndrome. It might be related to autoimmune temporal arteritis and its treatment with GCCs. Also, the exacerbation of temporal arteritis can cause ILD which was taken into account because of already existing diagnosis – temporal arteritis which was confirmed from the biopsy of temporal artery. Radiological findings can also show nonnecrotising granulomas which is a non-specific sign. Lung biopsy can help to exclude other possible lung injuries and confirm autoimmune processes in lungs.

## Conclusions

The current clinical case emphasizes issues which may be encountered by a physician differentiating amiodarone-induced lung toxicity with suspicion of interstitial lung disease or other lung diseases, like cancer or vasculitis. Firstly, amiodarone may cause non-necrotizing granulomatous changes in lungs when it is used 400 mg/daily or more for a prolonged period, as it was seen in the current case. Secondly, *Mycobacteria* may have low concentration in bodily fluids and it might not be diagnosed using Ziehl-Nielsen microscopy for AFB. Thirdly, the autoimmune ILD might be suspected when the patient has a medical history of any other granulomatous disease, as in this case – temporal arteritis which was treated with GCCs or any other immunosuppressive agents which had an impact on patient’s immune status and his susceptibility to bacterial infections.

## Abbreviations

ACEi: Angiotensin converting enzyme inhibitor
AF: Atrial fibrillation
AFB: Acid fast bacilli
BB: β blockers
DILD: Drug-induced interstitial lung disease
DIRD: Drug-induced respiratory disease
GCC: Glucocorticosteroids
HRCT: High resolution computed tomography
ICU: Intensive care unit
ILD: Interstitial lung disease
NICE: National Institute for Health and Clinical Excellence
OLB: Open lung biopsy
RF: Respiratory failure
TB: Tuberculosis
